# Microwave-assisted cross-linking of milk proteins induced by microbial transglutaminase

**DOI:** 10.1038/srep39040

**Published:** 2016-12-14

**Authors:** Chun-Chi Chen, Jung-Feng Hsieh

**Affiliations:** 1 Department of Food Science, Fu Jen Catholic University, Taipei 242, Taiwan; 2 Ph.D. Program in Nutrition & Food Science, Fu Jen Catholic University, Taipei 242, Taiwan

## Abstract

We investigated the combined effects of microbial transglutaminase (MTGase, 7.0 units/mL) and microwave irradiation (MI) on the polymerization of milk proteins at 30 °C for 3 h. The addition of MTGase caused the milk proteins to become polymerized, which resulted in the formation of components with a higher molecular-weight (>130 kDa). SDS-PAGE analysis revealed reductions in the protein content of β-lactoglobulin (β-LG), α_S_-casein (α_S_-CN), κ-casein (κ-CN) and β-casein (β-CN) to 50.4 ± 2.9, 33.5 ± 3.0, 4.2 ± 0.5 and 1.2 ± 0.1%, respectively. The use of MTGase in conjunction MI with led to a 3-fold increase in the rate of milk protein polymerization, compared to a sample that contained MTGase but did not undergo MI. Results of two-dimensional gel electrophoresis (2-DE) indicated that κ-CN, β-CN, a fraction of serum albumin (SA), β-LG, α-lactalbumin (α-LA), α_s1_-casein (α_s1_-CN), and α_s2_-casein (α_s2_-CN) were polymerized in the milk, following incubation with MTGase and MI at 30 °C for 1 h. Based on this result, the combined use of MTGase and MI appears to be a better way to polymerize milk proteins.

MTGase is widely used to modify the solubility, hydration, and heat stability of proteins in food products. MTGase can be used to catalyze the acyl transfer reaction between lysine residues and glutamine residues, which causes ε-(γ-glutamyl)lysine covalent bonds with intra- and inter-molecular cross-linking to form in proteins^1^,^2^. Among food proteins, milk proteins are particularly good substrates for MTGase. Rossa, de Sá, Burin, and Bordignon-Luiz^3^ claimed that the polymerization of milk proteins by MTGase improves the functional properties of dairy products. Agyare and Damodaran^4^ further reported that MTGase can be used to modify the heat stability of caseins as well as their gelation and renneting properties. Finally, MTGase has also been shown to improve the quality and yield of cheeses^5^.

Milk proteins include caseins (α_s1_-CN, α_s2_-CN, β-CN, and κ-CN) and whey proteins^6^. Casein micelles are particles that contain thousands of casein molecules and have a surface composed primarily of κ-CN^7^,^8^. Whey proteins are composed of SA, α-LA, and β-LG^9^. In a previous study, we determined that MTGase causes the polymerization of caseins and whey proteins; however, this process is time-consuming, requiring an incubation period of 3 h^10^.

MI has obvious advantages in certain enzymatic reactions^11^. Microwaves are part of the electromagnetic wave spectrum and have wavelengths ranging from 1 mm to 1 m. MI has been shown to enhance the yield and reaction rate in certain enzymatic reactions^12^. MI treatment has also been used to accelerate enzyme-catalyzed reactions in food products, thereby enhancing the activity and selectivity of glycosidases and decarboxylases^13^. In addition, Da Rós, Freitas, Perez and de Castro^14^ reported the application of lipase/MI treatment to accelerate the enzymatic synthesis of biodiesel, and high catalytic efficiency (*K*_cat_/*K*_m_) values have also been achieved in the microwave-assisted digestion of β-LG by pronase^15^. Finally, MI can be used in the MTGase catalysis of covalent cross-linking, and to improve the gel strength of soybean protein isolate gels^16^.

In this study, we investigated the application of MI to enhance the MTGase-induced polymerization of caseins and whey proteins. No previous research has used SDS-PAGE or 2-DE to elucidate the effects of microwave-assisted polymerization on milk molecules.

## Results and Discussion

### Effects of MI and MTGase on the polymerization of milk proteins

**Figure 1:**
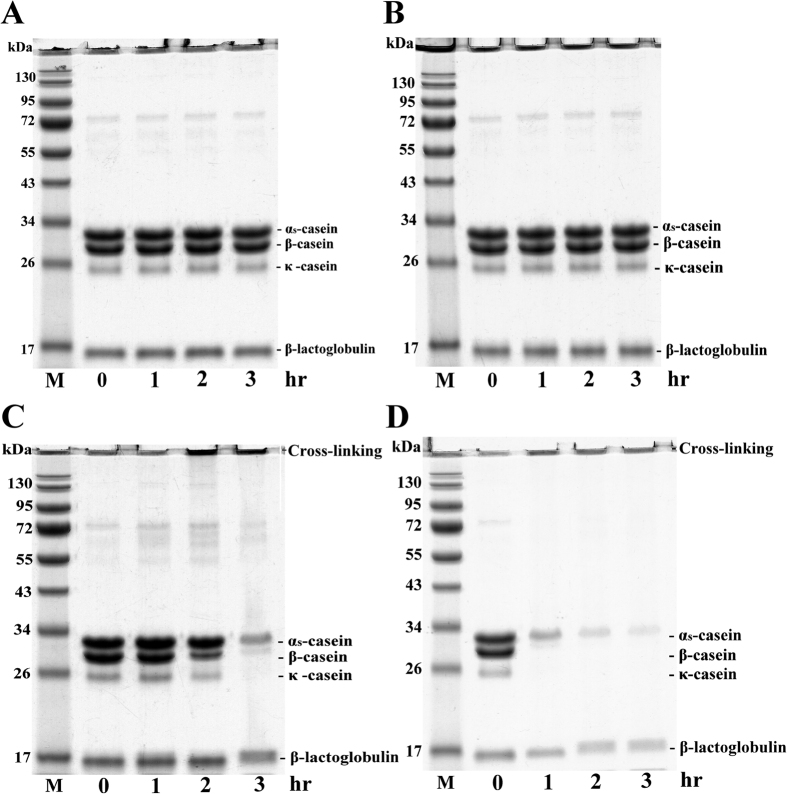
SDS-PAGE image showing milk samples incubated with/without MTGase and/or MI at 30 °C for 3 h: (**A**) Milk samples without MI; (**B**) milk samples with MI; (**C**) milk samples with MTGase (7.0 units/mL); and (**D**) milk samples with MTGase (7.0 units/mL) and MI.

We first investigated the effects of MI and MTGase (7.0 units/mL) on the polymerization of milk proteins (Fig. 1). Milk samples with/without MI were treated at 30 °C for 0, 1, 2, or 3 h prior to analysis by SDS-PAGE. No signs of polymerization were observed during this period, whether or not they underwent MI. Tyler-Cross and Schirch^17^ reported that the naturally occurring deamidation of protein is ubiquitous, and that it can affect both the function and structure of proteins. The rate of deamidation is affected by a number of factors, including the primary sequences of the proteins, temperature, pH, and the constituents of solutions. MI induces molecular rotation in the direction of dipole alignment by creating an external oscillating electric field. It is therefore reasonable to expect that MI can accelerate the deamidation of proteins. Liu, Moulton, Auclair, and Zhou^18^ developed a novel method for the study of protein deamidation using mass spectrometry, and it is very likely that their approach is also suitable for the study of milk proteins. In this study, milk samples incubated with MTGase at 30 °C for 3 h presented a reduction in the quantity of α_S_-CN, κ-CN, and β-CN (Fig. 1C). In agreement with our previous research, we observed that MTGase caused these proteins to be polymerized into higher-molecular-weight proteins (>130 kDa)^10^. Specifically, components with higher molecular weights were produced by casein (α_S_-CN, β-CN, and κ-CN) crosslinking. Smiddy, Martin, Kelly, de Kruif, and Huppertz^19^ reported that MTGase is an acyltransferase capable of inducing intra- and intermolecular crosslinking reactions involving milk proteins. Thus, the disappearance of caseins suggests that MTGase-induced polymerization occurred. Liu *et al*.^20^,^21^ developed a method that uses ^18^O-labeling and mass spectrometry to identify protein cross-linking. Their strategy, which is based on XChem-Finder, can be used to identify cross-linking in individual caseins.

Molecules with high dipole moments, such as enzymes and proteins, are particularly susceptible to the effects of microwaves^22^,^23^. Indeed, MI has been shown to accelerate MTGase-polymerized reactions in milk samples; therefore, we propose that MI could also be used to induce enzymatic reactions involving MTGase. In this study, the use of MTGase in conjunction with MI for 1 h led to a 3-fold increase in the rate of milk protein polymerization, compared to a sample that contained MTGase but did not undergo MI (Fig. 1D). These results corroborate findings from previous works. For example, Huang *et al*.^11^ used MI to increase the rates of reactions involved in the esterification between *n*-caprylic acid and pentanol isomers catalyzed via lipase. Wan, Sun, Hu, and Xia^24^ suggested that MI has nonthermal effects on enzymatic reactions, which is primarily due to the polarities of the substrates and solvents. Young, Nichols, Kelly, and Deiters^25^ reported that MI is an effective tool for increasing the reaction rates in the activation of enzymatic catalysis.

**Figure 2:**
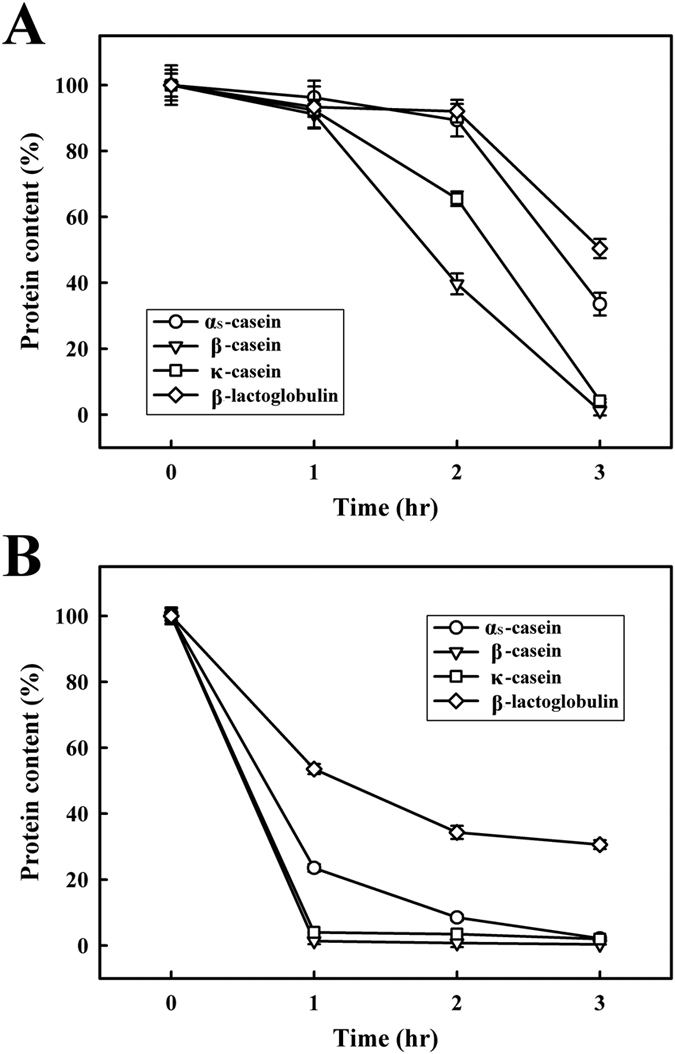
SDS-PAGE analysis of caseins and whey proteins treated with MTGase under MI at 30 °C for 3 h. (**A**) Milk samples incubated with MTGase (7.0 units/mL) and (**B**) milk samples incubated with MTGase (7.0 units/mL) under MI.

Figure 2 illustrates the SDS-PAGE analysis of caseins and whey proteins incubated with MTGase under MI at 30 °C. As shown in Fig. 2A, no polymerization occurred after 1 h of incubation; however after 3 h, the protein contents of β-LG, α_S_-CN, κ-CN and β-CN were reduced to 50.4 ± 2.9, 33.5 ± 3.0, 4.2 ± 0.5 and 1.2 ± 0.1%, respectively. These results indicate that the MTGase-induced polymerization of κ-CN, β-CN, and α_S_-CN occurred earlier than that of β-LG. De Jong and Koppelman^26^ previously reported that β-LG is a poor substrate for MTGase. In this study, after being incubated at 30 °C for 1 h, the protein content of β-LG, β-CN, κ-CN, and α_S_-CN was noticeably lower in samples where MTGase was used in conjunction with MI than in samples which contained MTGase but did not undergo MI (Fig. 2B). After incubation at 30 °C for 1 h, the protein content of β-LG, α_S_-CN, κ-CN, and β-CN decreased to 30.6 ± 1.3, 2.1 ± 0.7, 1.9 ± 0.2, and 0.3 ± 0.1%, respectively. Bornaghi and Poulsen^27^ reported the use of MI to accelerate enzymatic processes. Our results indicate that MI treatment can be used to accelerate the MTGase-polymerized reactions of caseins and whey proteins.

### 2-DE analysis of MTGase and MI treated milk proteins

**Figure 3:**
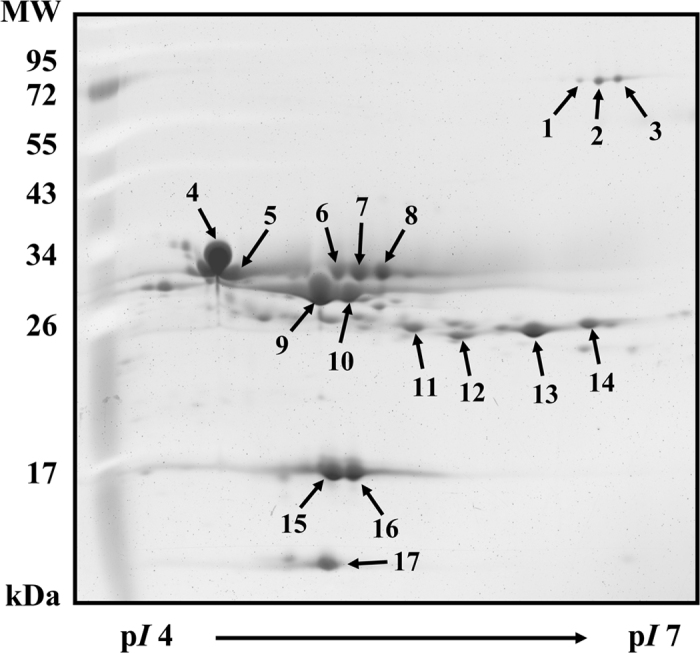
2-DE image showing a milk sample incubated without MTGase or MI.

**Figure 4:**
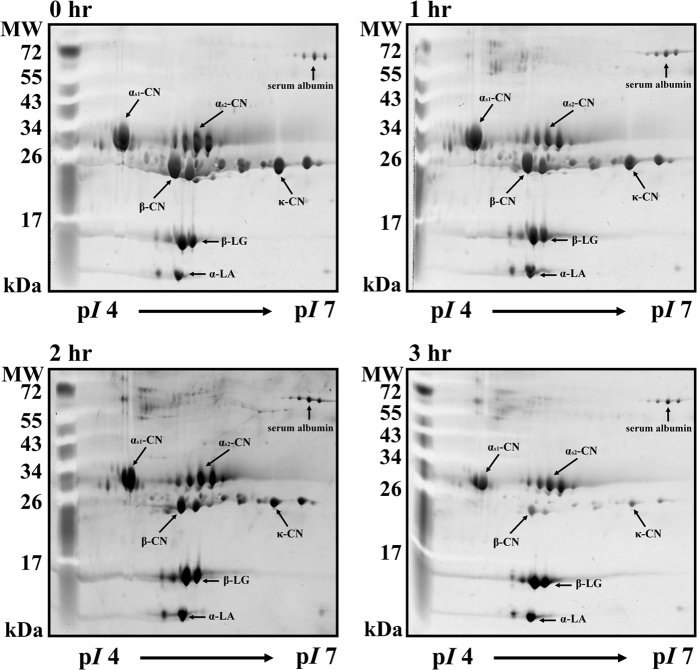
2-DE image showing milk samples after treatment with MTGase (7.0 units/mL) at 30 °C for 3 h.

**Figure 5:**
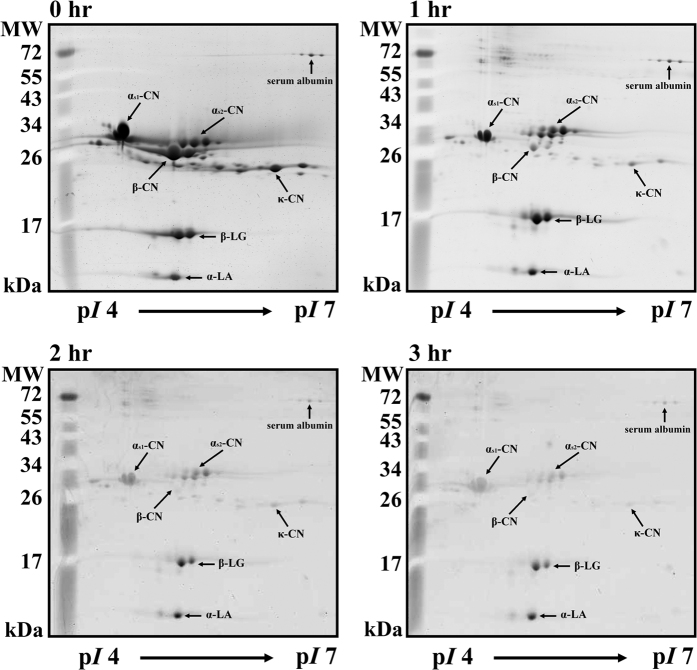
2-DE image showing milk samples after treatment with MI and MTGase (7.0 units/mL) at 30 °C for 3 h.

As shown in Fig. 3, 17 milk proteins were observed on the 2-DE gel, all of which were identified in our previous study^28^. Numbers (No.) 1 to 17 on the 2-DE gel represent SA (No. 1–3), α_s1_-CN (No. 4–5), α_s2_-CN (No. 6–8), β-CN (No. 9–10), κ-CN (No. 11–14), β-LG (No. 15–16), and α-LA (No. 17). α-LA, SA, and β-LG are components of whey proteins, whereas κ-CN, β-CN, α_s1_-CN, and α_s2_-CN are components of caseins^29^. Milk samples were incubated with MTGase (7.0 units/mL) at 30 °C for 3 h prior to analysis by 2-DE (Fig. 4). We found that α_s1_-CN (No. 4–5), α_s2_-CN (No. 6–8), β-CN (No. 9–10), and κ-CN (No. 11–14) as well as portions of SA (No. 1–3), β-LG (No. 15–16) and α-LA (No. 17) showed signs of depletion after incubation for 3 h in the MTGase-containing milk. This indicates that most of the casein micelles and a portion of the whey proteins were polymerized by MTGase. We also observed that the casein polymerization of α_s1_-CN and α_s2_-CN was slower than that of β-CN and κ-CN. Smiddy, Martin, Kelly, de Kruif, and Huppertz^19^ reported that κ-CN and β-CN molecules are more readily catalyzed by MTGase. In this study, the application of MI for 1 h substantially improved the MTGase-induced polymerization of milk proteins. Specifically, α_s1_-CN (No. 4–5), α_s2_-CN (No. 6–8), β-CN (No. 9–10), and κ-CN (No. 11–14), as well as portions of SA (No. 1–3), β-LG (No. 15–16), and α-LA (No. 17) showed signs of depletion after incubation for 1 h in the MTGase-containing milk (Fig. 5). Roy and Gupta^30^ observed that MI increased the rate of initial enzymatic reactions at all levels of (trans-) esterification.

**Figure 6:**
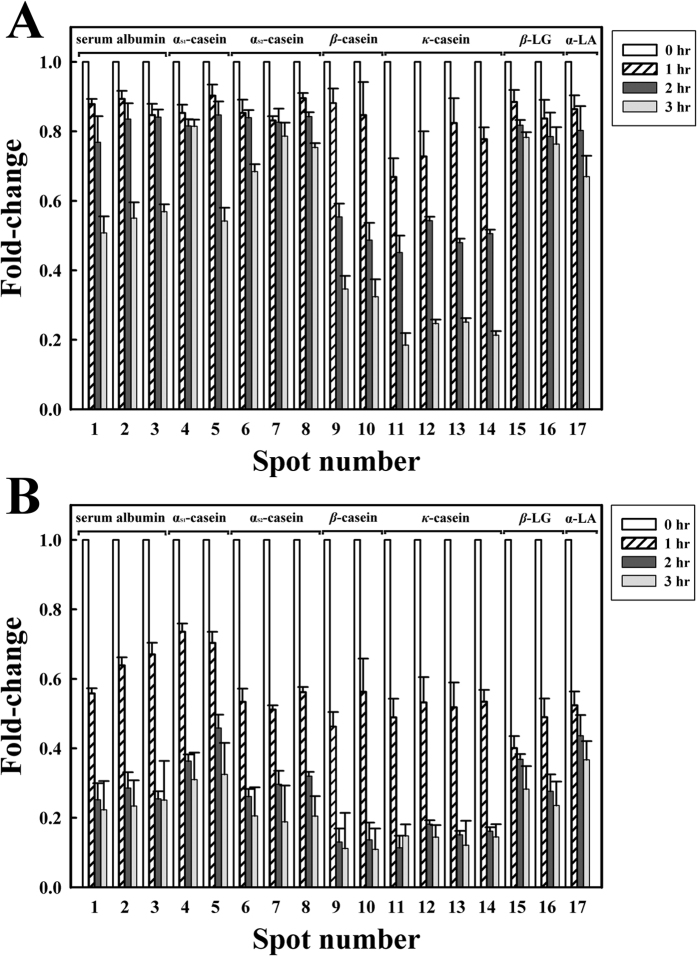
Relative abundance of caseins and whey proteins after treatment with MTGase and MI at 30 °C for 3 h. (**A**) Milk samples treated with MTGase (7.0 units/mL) and (**B**) milk samples treated with MTGase (7.0 units/mL) and MI. The protein numbers correspond to those in Fig. 3.

Relative abundance of caseins and whey proteins after treatment with MTGase and MI are shown in Fig. 6. The fold changes of the milk proteins in the MTGase-containing milk decreased significantly after 3 h of incubation (Fig. 6A). The changes in No. 1–17 in the MTGase-containing milk were 0.50-, 0.55-, 0.56-, 0.81-, 0.54-, 0.68-, 0.78-, 0.75-, 0.34-, 0.32-, 0.18-, 0.24-, 0.25-, 0.21-, 0.78-, 0.76-, and 0.67-fold, respectively. Our analysis revealed that caseins were partially polymerized by MTGase following 3 h of incubation, including 19% of α_s1_-CN in No. 4, 46% of α_s1_-CN in No. 5, 32% of α_s2_-CN in No. 6, 22% of α_s2_-CN in No. 7, 25% of α_s2_-CN in No. 8, 66% of β-CN in No. 9, 68% of β-CN in No. 10, 82% of κ-CN in No. 11, 76% of κ-CN in No. 12, 75% of κ-CN in No. 13, and 79% of κ-CN in No. 14. Whey proteins were also partially polymerized after 3 h of incubation, including 50% of SA in No. 1, 45% of SA in No. 2, 44% of SA in No. 3, 22% of β-LG in No. 15, 24% of β-LG in No. 16, and 33% of α-LA in No. 17.

Figure 6B illustrates the 2-DE analysis of milk samples incubated with MTGase under MI. This analysis revealed that 45% of SA in No. 1, 37% of SA in No. 2, 33% of SA in No. 3, 27% of α_s1_-CN in No. 4, 30% of α_s1_-CN in No. 5, 47% of α_s2_-CN in No. 6, 49% of α_s2_-CN in No. 7, 44% of α_s2_-CN in No. 8, 54% of β-CN in No. 9, 44% of β-CN in No. 10, 52% of κ-CN in No. 11, 47% of κ-CN in No. 12, 49% of κ-CN in No. 13, 47% of κ-CN in No. 14, 60% of β-LG in No. 15, 52% of β-LG in No. 16, and 48% of α-LA in No. 17 were polymerized after 1 h of incubation. After 3 h of incubation, these proteins had undergone near complete polymerization.

### Effects of incubation condition on MTGase activity in buffer and milk samples

**Figure 7:**
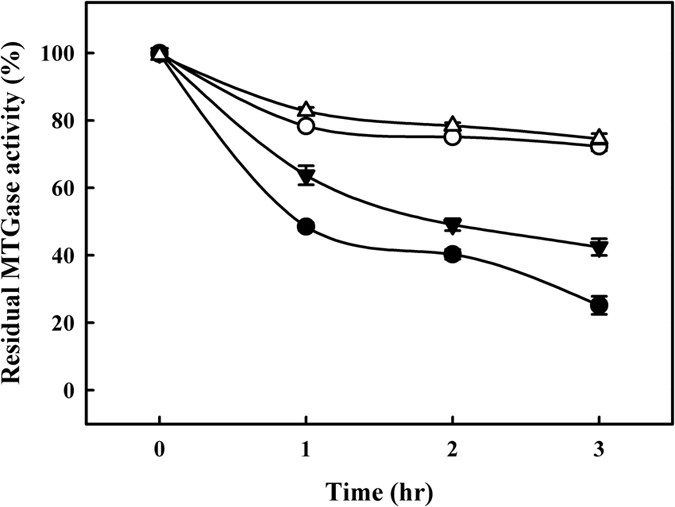
Effects of incubation time on MTGase activity: (△) MTGase-containing buffer with MI; (○) MTGase-containing buffer without MI; (▼) MTGase-containing milk samples with MI; and (●), MTGase-containing milk samples without MI.

MTGase-containing buffer (0.1 M Tris-acetate buffer, pH 6.0) was incubated in a dry bath at 30 °C for 3 h. Residual MTGase activity in the buffer decreased slightly as incubation time increased (Fig. 7). After 3 h of incubation, the residual MTGase activity had decreased to 72.3 ± 1.4% of the original level. Similar trends were observed for residual MTGase activity in the MTGase-containing buffer when MI was performed at 30 °C for 3 h. Specifically, residual MTGase activity decreased to 74.5 ± 1.5% after 3 h of incubation. Jiang, Hsieh, and Tsai^31^ also reported a reduction in residual MTGase activity (to 42.1 ± 2.9%) in MTGase-containing buffer (50 mM sodium phosphate buffer, pH 7.0) when samples were incubated at 45 °C for 2 h. Ho, Leu, Hsieh, and Jiang^32^ reported that the rate constant (*K*_D_) for the thermal inactivation of MTGase at 45 °C was 6.2 × 10^−5^/min. In addition, the residual MTGase activity in MTGase-containing milk was 99.8 ± 0.8% when the sample was incubated in a dry bath incubator at 30 °C for 0 h. These results suggest that the components of milk can not affect the activity of MTGase. Nonetheless, the residual MTGase activity in milk decreased significantly to 25.2 ± 2.7% after 3 h of incubation. Therefore, the residual MTGase activity in milk samples incubated at 30 °C for 3 h was lower than that in buffer samples (*P* < 0.05). As previously mentioned, we observed that caseins and whey proteins were polymerized into higher-molecular-weight proteins by MTGase, which means that MTGase was probably entrapped within the high-molecular-weight polymers, thereby leading to a reduction in residual MTGase activity.

MTGase-containing milk samples were also incubated in a microwave reactor at 30 °C for 3 h, which resulted in a significant decrease in residual MTGase activity to just 42.4 ± 2.5% (*P* < 0.05). The residual MTGase activity in all milk samples incubated in the microwave reactor exceeded that of the samples incubated in the dry bath incubator. This indicates that MTGase is more stable under MI, and these findings are supported by Yu *et al*.^33^ who reported that the lipase Novozym 435 is more stable under MI than under conventional heating. Réjasse, Lamare, Legoy, and Besson^34^ proposed that MI enhances interactions between chemical bonds involved in maintaining the enzymatic structure, thereby protecting enzymes from thermal denaturation.

### Catalytic procedure for the microwave-assisted polymerization of milk proteins induced by MTGase

**Figure 8:**
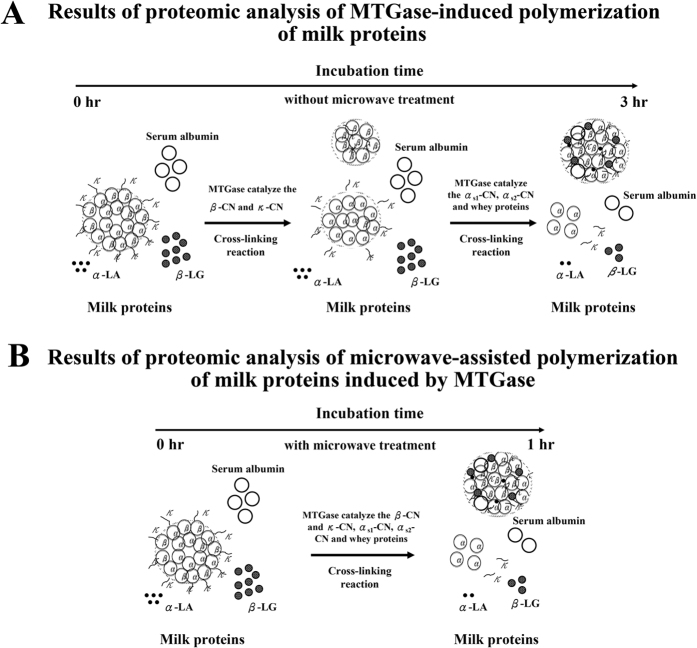
Catalytic procedure for the microwave-assisted polymerization of milk proteins induced by MTGase: (**A**) Milk samples with MTGase (7.0 units/mL) and (**B**) milk samples with MTGase (7.0 units/mL) and MI.

Figure 8 presents the microwave-assisted catalytic procedure applied to the polymerization of milk proteins by MTGase. The MTGase-induced polymerization of milk proteins was observed after 3 h of incubation (Fig. 8A). MTGase catalyzes the cross-linking of κ-CN and β-CN before catalyzing SA, α-LA, α_s1_-CN, α_s2_-CN, and β-LG. The degree of β-CN and κ-CN polymerization exceeded that of SA, α-LA, α_s1_-CN, α_s2_-CN, and β-LG. De Kruif and Holt^35^ reported that MTGase-induced polymerization occurs more readily for β-CN and κ-CN than for other milk proteins, which may be an effect of the specific location of MTGase within the casein micelle. Sharma, Lorenzen, and Qvist^36^ reported that the porous nature of casein micelles allows MTGase to diffuse into the interior. This means that the flexible conformation of β-CN in casein micelles also allows for catalysis by MTGase. In the current study, MI treatment was found to accelerate the MTGase-polymerized reactions in milk samples, wherein the MTGase-induced polymerization of milk proteins was observed after just 1 h of incubation (Fig. 8B). Within 1 h, most of the κ-CN and β-CN as well as fractions of SA, α-LA, β-LG, α_s2_-CN, and α_s1_-CN had been polymerized by MTGase into higher-molecular-weight proteins. Thus, the polymerized milk proteins contained κ-CN, β-CN, SA, β-LG, α-LA, α_s2_-CN, and α_s1_-CN fractions.

In summary, we examined the effects of MTGase and MI on the polymerization of individual proteins in milk. After 3 h of incubation, the MTGase had polymerized κ-CN, β-CN and a portion of SA, α_s1_-CN, α_s2_-CN, α-LA, and β-LG fractions to form high-molecular-weight polymers. Furthermore, the polymerization of SA, β-LG, α-LA, α_s2_-CN, and α_s1_-CN was slower and occurred earlier than the polymerization of β-CN and κ-CN. The use of MTGase in conjunction with MI for 1 h substantially improved the polymerization of caseins and whey proteins, indicating that MI accelerates MTGase-polymerized reactions in milk.

## Materials and Methods

### Preparation of MTGase and milk samples

Milk was first centrifuged at 5,000 × g for 20 min, and a layer of fat was subsequently removed from the top of the milk using a spatula. The resulting skimmed milk was then stored at 4 °C. A Bio-Rad protein assay kit was used in accordance with manufacturer’s instructions to determine the protein concentrations in the skimmed milk, using SA as a standard. MTGase from *Streptoverticillium mobaraense* (1.0 units/mg) was purchased from Ajinomoto Co. Inc. (Activa TG-B, solution form; Ajinomoto Co. Inc., Tokyo, Japan). All experiments on the microwave-assisted polymerization of milk proteins were performed using 1 mL milk samples contained within a closed vessel, where they did not undergo any stirring or shaking. To prepare milk with/without MTGase (7.0 units/mL), we first incubated the milk samples in a dry bath incubator at 30 °C for 0, 1, 2 and 3 h, respectively. The resulting samples were then heated to 80 °C for 3 min to deactivate the MTGase. The microwave-assisted polymerization of milk proteins induced by MTGase was investigated using a microwave reactor (CEM^®^ Discover system, CEM Corporation, Matthews, NC, USA). Milk samples with/without MTGase (7.0 units/mL) were incubated in the microwave reactor at 30 °C at a power of 30W for 0, 1, 2, or 3 h. Samples were then heated to 80 °C for 3 min to deactivate the MTGase. Experiments were conducted under one of four conditions: (1) milk without MTGase or MI; (2) milk with MTGase; (3) milk with MI; and (4) milk with MTGase and MI.

### SDS-PAGE and 2-DE analysis

Milk samples were analyzed using SDS-PAGE and 2-DE, in accordance with protocol outlined by Hsieh and Pan^10^. For SDS-PAGE analysis, milk samples were analyzed using 12.5% separating gels. Milk samples were mixed with loading buffer before being denatured at 100 °C for 5 min. Five μL of the milk samples with protein markers (10–200 kDa) were loaded onto the SDS-PAGE gel, and separation was performed at 90 V in the separating gel. Following electrophoresis, Coomassie Brilliant Blue R-250 staining was used for the detection of protein bands. Gel images were then scanned and analyzed using Gel-Pro Analyzer software. For 2-DE analysis, milk proteins (100 μg) were solubilized in rehydration buffer to a final sample volume of 350 μL. The IPG gel strips were then focused on the soluble proteins. For this, we used 12.5% separating gels in the second dimension. Each 2-DE gel was stained and scanned using the Typhoon 9200 imaging system. Finally, we used Progenesis SameSpots software to analyze gel images.

### Determination of MTGase activity

MTGase (7.0 units/mL) was respectively added to buffer (0.1 M Tris-acetate buffer, pH 6.0) and milk samples. The MTGase-containing buffer and milk samples were incubated in a dry bath incubator or microwave reactor (CEM^®^ Discover system, power of 30 W) at 30 °C for 0, 1, 2, or 3 h. This experiment was conducted under one of four conditions: (1) MTGase-containing buffer without MI; (2) MTGase-containing buffer with MI; (3) MTGase-containing milk without MI; and (4) MTGase-containing milk with MI. The resulting samples were then analyzed using MTGase assay to assess residual MTGase activity in accordance with the method proposed by Ho, Leu, Hsieh, and Jiang^32^.

### Statistical analysis

SPSS statistical software was used for statistical analysis. The results are presented as mean ± standard deviation. Duncan’s multiple range tests and one-way ANOVA were used to calculate significant differences between treatments. Three determinations were performed for each treatment, and the level of statistical significance was set at *P* < 0.05.

## Additional Information

**How to cite this article**: Chen, C.-C. and Hsieh, J.-F. Microwave-assisted cross-linking of milk proteins induced by microbial transglutaminase. *Sci. Rep.* **6**, 39040; doi: 10.1038/srep39040 (2016).

**Publisher's note:** Springer Nature remains neutral with regard to jurisdictional claims in published maps and institutional affiliations.
